# Predictive analysis methods for human microbiome data with application to Parkinson’s disease

**DOI:** 10.1371/journal.pone.0237779

**Published:** 2020-08-24

**Authors:** Mei Dong, Longhai Li, Man Chen, Anthony Kusalik, Wei Xu

**Affiliations:** 1 Dalla Lana School of Public Health, University of Toronto, Toronto, ON, Canada; 2 Department of Mathematics and Statistics, University of Saskatchewan, Saskatoon, SK, Canada; 3 Department of Computer Science, University of Saskatchewan, Saskatoon, SK, Canada; 4 Department of Biostatistics, Princess Margaret Hospital, Toronto, ON, Canada; Indiana University School of Medicine, UNITED STATES

## Abstract

Microbiome data consists of operational taxonomic unit (OTU) counts characterized by zero-inflation, over-dispersion, and grouping structure among samples. Currently, statistical testing methods are commonly performed to identify OTUs that are associated with a phenotype. The limitations of statistical testing methods include that the validity of p-values/q-values depend sensitively on the correctness of models and that the statistical significance does not necessarily imply predictivity. Predictive analysis using methods such as LASSO is an alternative approach for identifying associated OTUs and for measuring the predictability of the phenotype variable with OTUs and other covariate variables. We investigate three strategies of performing predictive analysis: (1) LASSO: fitting a LASSO multinomial logistic regression model to all OTU counts with specific transformation; (2) screening+GLM: screening OTUs with q-values returned by fitting a GLMM to each OTU, then fitting a GLM model using a subset of selected OTUs; (3) screening+LASSO: fitting a LASSO to a subset of OTUs selected with GLMM. We have conducted empirical studies using three simulation datasets generated using Dirichlet-multinomial models and a real gut microbiome data related to Parkinson’s disease to investigate the performance of the three strategies for predictive analysis. Our simulation studies show that the predictive performance of LASSO with appropriate variable transformation works remarkably well on zero-inflated data. Our results of real data analysis show that Parkinson’s disease can be predicted based on selected OTUs after the binary transformation, age, and sex with high accuracy (Error Rate = 0.199, AUC = 0.872, AUPRC = 0.912). These results provide strong evidences of the relationship between Parkinson’s disease and the gut microbiome.

## Introduction

The microbiome comprises all of the genetic material within a microbiota (the entire collection of microorganisms in a specific niche, such as the human gut). This can also be referred to as the metagenome of the microbiota [1]. The far-reaching effects of the microbiome on human diseases and many other biological phenotypes have only recently been discovered [2]. Bacteria in the body and on its surface have a significant impact on the development of health and disease states. For example, microbial changes are shown to be associated with Parkinson’s disease [3]. The abundance of a bacterium species or genus is quantified by OTU counts using genetic sequence similarity, produced via targeted amplification and sequencing of the 16S rRNA gene [4].

OTU counts often have number of zeros more than what is expected in Poisson or negative binomial (NB) models. One source of the zero microbiota abundance is that only a few major bacterial taxa of the microbiota are shared across samples and the rest are detected only in a small percentage of the samples. The zero counts may also be observed when the counts are present with a low frequency but not observed because of sampling variation (sampling zeros). When OTU counts are non-zero, it is often observed that they are highly right skewed, often called over-dispersion. Many studies have been developed to account for the over-dispersion of microbiome data such as logistic normal multinomial regression [5] and Dirichlet-multinomial regression [6]. However, those methods do not consider the grouping structure of microbiome data; for example, plants from the same plot, and individuals from the same family. The grouping structure in the sample causes correlation among the samples and thus further complicates the analysis and interpretation of microbiome count data. Ignoring the correlation among samples can result in biased inference and misleading results. Generalized linear mixed effect models (GLMMs) are often adopted to account for the grouping structure by treating the group identities as random factors [7, 8].

It is of great interest to find the association between the abundances of a subset of OTUs and a host factor, such as a health disorder; see [9–11]. For example, Huttenhower et al. [12] report various relationships between the gut microbiome and cancer, inflammatory bowel disease, and obesity. Currently, researchers fit each OTU variable with GLMM given the phenotype variable and other factors, and then apply a statistical testing method to each OTU to test whether the OTU is differentiated by the phenotype variable [7, 8]. OTU selection can be achieved by thresholding the p-values/q-values returned by the statistical testing procedure. However, statistical testing methods have a number of limitations. First, the validity of p-values/q-values relies on the correctness of assumed models, which may not hold for real datasets. Second, p-values/q-values only measure statistical significance but not practical significance. Small q-values do not necessarily imply strong predictivity. For example, many SNPs selected by genome-wide association studies are not good predictors [13–15].

Considering the limitations of statistical testing methods, we are interested in performing predictive analysis for microbiome data. We employ statistical machine learning methods to make predictions of a phenotype based on microbial composition and other covariates. The strength of the association between microbial composition and the phenotype is measured by predictive metrics such as error rate, AUC, etc. In this paper, we consider fitting a logistic regression model for a phenotype variable given microbial composition and other covariates. Due to the large number of OTUs, which is often larger than the sample size, a penalization for the regression coefficients is necessary for controlling over-fitting, for which LASSO [16] is a popular choice. LASSO uses a *L*_1_ penalization that can shrink some coefficients to exactly 0 for OTU selection and for controlling over-fitting. To the best of our knowledge, fitting the distribution of a phenotype given OTU composition with a method such as LASSO has not been adopted very often by researchers in microbiome studies. Statistical methods based on fitting GLMM models for OTU counts given a phenotype and other covariates are typically used for identifying the association between microbial composition and a phenotype; see [7, 8]. The primary advantage of fitting a model for a phenotype given OTU composition data is that this approach can capture the joint effect of OTU composition on the phenotype. Statistical testing methods that model each OTU individually [17–19] may fail to discover such a complex relationship. For example, a phenotype may be associated with the proportion of a microbial taxon rather than individual OTUs within the taxon; in such a scenario, each OTU within the taxon may exhibit weak association with the phenotype.

In this paper, we emphasize that classical statistical testing method is not enough for microbiome data. Strong association does not imply good predictive performance. Moreover, wrongly specified model can give biased results. Simple statistical learning method such as LASSO with appropriate transformation works well for microbiome data. We combine the statistical testing and learning method for microbiome data. We consider three strategies of predictive analysis for microbiome data: (1) fitting a LASSO multinomial logistic regression (LASSO-MLR) model to all OTU counts with specific transformation, shortened by **LASSO**; (2) screening OTUs with q-values returned by fitting a GLMM to each OTU, then fitting a GLM model using a subset of selected OTUs, shortened by **screening+GLM**; (3) fitting a LASSO-MLR to a subset of OTUs selected with GLMM, shortened by **screening+LASSO**. In the meantime, we investigate the predictive effect of random effect on phenotypes. We have conducted empirical studies using synthetic datasets and a real gut microbiome data related to Parkinson’s disease (PD) to investigate the performance of the three strategies for predictive analysis. Three synthetic datasets were generated using Dirichlet-multinomial model. Then phenotypes are generated using logistic regression given OTUs, fixed and random effects. For OTU screening, we apply the likelihood ratio test (LRT) to three GLMMs that can handle zero-inflation and over-dispersion of OTU counts.

Our studies with the synthetic datasets show that the predictive performance of LASSO without variable screening is remarkably excellent for zero-inflated data. The screening+GLM and screening+LASSO methods also work reasonably well if the power of screening is high. The analysis also shows that random effects can help to improve the predictive accuracy. The applications of the three strategies of predictive analysis methods in the PD microbiome dataset show that LASSO can predict PD with microbial composition and two covariates (age and sex) accurately with error rates near 0.2 and AUCs higher than 0.8. The best predictive accuracy for PD with this dataset is obtained with a screening+LASSO method, which gives predictive metrics as follows: ER = 0.199, AUC = 0.872, AUPRC = 0.912. Our predictive analysis results provide strong evidences of the relationship between PD and the gut microbiome.

## Methods

### Notations for micorbiome data

A typical OTU dataset contains measurements of abundance for OTUs, the total reads, a number of fixed factors and random factors for each sample, as shown by Table 1:

**Table 1:** A general form of microbiome data.

	OTU_1_	⋯	OTU_*m*_	Total reads	Phenotype	Fixed factors	Random factors
Sample 1	$Z_{1}^{\left(1\right)}$	⋯	$Z_{1}^{\left(m\right)}$	*T*_1_	*Y*_1_	$X_{1}^{\left(1\right)},\cdots ,X_{1}^{\left(s\right)}$	$W_{1}^{\left(1\right)},\cdots ,W_{1}^{\left(t\right)}$
⋮	⋮	⋯	⋮	⋮	⋮	⋮	⋮
Sample *n*	$Z_{n}^{\left(1\right)}$	⋯	$Z_{n}^{\left(m\right)}$	*T*_*n*_	*Y*_*n*_	$X_{n}^{\left(1\right)},\cdots ,X_{n}^{\left(s\right)}$	$W_{n}^{\left(1\right)},\cdots ,W_{n}^{\left(t\right)}$

We will describe the mathematical details of the notations in Table 1:

- $Z_{i}^{\left(j\right)}$, *i* ∈ [1, *n*], *j* ∈ [1, *m*] is the count of OTU *j* in sample *i*. This number can be the abundance of taxa grouped at different levels such as species, genus, and family.
- *T*_*i*_, *i* ∈ [1, *n*] is the total number of sequence reads for sample *i*. If all measured OTUs are included in our model, $T_{i}=\sum_{j=1}^{m}Z_{i}^{\left(j\right)}$. However, *T*_*i*_ may be smaller than $\sum_{j=1}^{m}Z_{i}^{\left(j\right)}$ if some OTUs are omitted for measurement quality control reasons.
- *Y*_*i*_, *i* ∈ [1, *n*] is the phenotype of interest.
- $X_{i}^{\left(j\right)}$, *i* ∈ [1, *n*], *j* ∈ [1, *s*] represents the *j*^*th*^ fixed factor associated with the *i*^*th*^ sample. *s* is the total number of fixed factors considered in the mixed effect model. They could be host or clinical factors. When the *j*th fixed factor is a categorical variable with *k* classes, $X_{i}^{\left(j\right)}$ is a row vector of *k* − 1 binary variables indicating the class of sample *i*.
- $W_{i}^{\left(j\right)}$, *i* ∈ [1, *n*], *j* ∈ [1, *t*] represents the *j*^*th*^ random factor associated for the *i*^*th*^ sample. *t* is the number of random factors considered in the mixed effect model. They are used to account for the correlations between samples since microbiota from the same group of samples are more similar than the ones from different groups. Similar to $X_{i}^{\left(j\right)}$, the $W_{i}^{\left(j\right)}$ is a row vector of binary indicator variables to represent the group identity of sample *i* in the *j*th random factor.

An important goal of microbiome studies is to identify a subset of OTUs that are associated with a phenotype *Y*, for example, a variable indicating disease status or plant traits. A typical way is to treat *Y* as a fixed factor and to fit each OTU variable *Z*^(*j*)^ with GLMM given all fixed and random factors, and then apply a statistical testing method to test whether the relative abundance of the *j*th OTU, which is defined as $Z_{i}^{\left(j\right)}/T_{i}$, is differentiated (i.e., associated) with *Y* [7, 8]. Selection of OTUs can be achieved by thresholding the q-values returned by the statistical testing procedure. Using statistical learning methods is another important alternative approach to selecting OTUs. In statistical learning methods, the phenotype *Y* is treated as a response variable; the predictor variables are OTU variables *Z*^(1)^, …, *Z*^(*m*)^ after transformation and some fixed and random factors. Selection of OTUs can be achieved by looking at the coefficients associated with OTU variables and the strength of the association can be measured with predictive metrics such as error rate and AUC. Different from using all OTU variables *Z*^(1)^, …, *Z*^(*m*)^ as predictors, another approach is to do feature selection twice by fitting LASSO after implementing statistical testing methods. This approach requires us to perform a statistical testing method on each OTU and select a subset of top OTUs by thresholding q-values. Then we can fit a LASSO MLR with the selected subset of OTUs and other factors as predictors. The performance of these three distinct methods for feature selection will be compared using synthetic datasets and a real gut microbiome dataset.

### Variable screening with GLMMs

#### GLMMs for zero-inflated data

GLMM is a flexible modelling framework that can take into account both fixed effects and random effects into the modelling of a response variable. GLMM is an extension of generalized linear models (GLMs) [20]. In this section, we will describe three GLMMs which are often used to model count data with zero-inflation and over-dispersion. We will apply GLMMs to model each OTU variable $Z_{i}^{\left(j\right)}$ individually, conditional on fixed and random factors as shown in Table 1. For simplicity in notations, we will omit the OTU index *j* throughout in this section.

In a NB mixed model, we use a NB distribution to model *Z*_*i*_ given fixed and random factors. The probability mass function (PMF) of *Z*_*i*_ is given by:

$$\begin{array}{c} f^{\text{NB}}\left(z_{i};\mu_{i},\theta \right)=\frac{\Gamma (z_{i}+\theta )}{\Gamma \left(\theta \right)\Gamma \left(z_{i}+1\right)}{\left(\frac{\theta }{\theta +\mu_{i}}\right)}^{\theta }{\left(\frac{\mu_{i}}{\theta +\mu_{i}}\right)}^{z_{i}}, \end{array}$$ (1)

where *z*_*i*_ takes values in {0, 1, …}, *μ*_*i*_ > 0 is the mean of *z*_*i*_, and *θ* > 0 is the inverse dispersion parameter. In NB mixed models, the mean *μ*_*i*_ is linked to fixed factors and random factors as follows:

$$\begin{array}{c} \log\left(\mu_{i}\right)=\log\left(T_{i}\right)+X_{i}\beta +W_{i}b \end{array}$$ (2)

where $X_{i}=\left(X_{i}^{\left(1\right)},\ldots ,X_{i}^{\left(s\right)}\right)$ is a vector representing all fixed factors, and similarly $W_{i}=\left(W_{i}^{\left(1\right)},\ldots ,W_{i}^{\left(t\right)}\right)$ is a vector of binary dummy variables representing all random factors. The total reads *T*_*i*_ exhibit big differences across samples. Thus, the log of total reads *T*_*i*_ is also considered as a random factor and added to the link function with fixed coefficient 1. Such a variable is often called the offset variable. In other words, Eq (2) links the log of *μ*_*i*_/*T*_*i*_—the proportion of the abundance of an OTU among all OTUs—to fixed and random factors.

The NB distribution has heavier tails than the Poisson distribution. When *θ* → ∞, the NB distribution converges to Poisson distribution. Compared to other distributions such as Poisson or normal, the advantage of using the NB distribution with small parameter *θ*, such as 1 or 2, is that the heavier tails of NB can reduce the influence of extraordinarily large (over-dispersed) counts *Z*_*i*_ in estimating the parameters in *μ*_*i*_. Other characteristics of microbiome data are the presence of many zeros. To address the zero-inflation in *Z*_*i*_, two-part models and zero-inflated models have been adopted for modelling *Z*_*i*_.

In two-part models, the modeling stage is divided into two parts. The first part models the presence-absence outcome via a binary model. The probability that *z*_*i*_ = 0 is modified by value *ϕ*_*i*_ and *ϕ*_*i*_ is often linked to fixed and random factors using logistic regression. The second part models the positive outcomes through a zero-truncated model; for example, a zero-truncated Poisson or NB distribution. This is also called hurdle model. Here we consider a more comprehensive two-part NB (TPNB) model based on the zero proportion (ZP) of each OTU [11]: (a) when ZP ≤ 10%, we fit the data based on NB model; (b) when 10%≤*ZP* ≤ 80%, we fit the data based on NB hurdle model; (c) when 80%≤*ZP* ≤ 90%, we fit the data based on logistic regression model; (d) OTU is dropped from the analysis if ZP ≥ 90%.

Another way to model zero-inflated count data is to use a zero-inflated model, which is modeling *Z*_*i*_ as a mixture distribution of 0 and a standard distribution such as Poisson or NB [21], instead of using a zero-truncated distribution for non-zero *z*_*i*_. Zero-inflated model is often understood as *z*_*i*_ being generated in two steps. The first step is to generate a binary indicator from a logistic regression distribution. In the second step, if the indicator in the first step is zero, then the *z*_*i*_ is zero; otherwise, *z*_*i*_ is generated from a standard distribution such as NB. In particular, the PMF of a zero-inflated negative binomial (ZINB) model for *Z*_*i*_ can be written as:

$$\begin{array}{c} f^{\text{ZINB}}\left(z_{i}\right)=\{\begin{array}{cc} \phi_{i}+\left(1-\phi_{i}\right)f^{\text{NB}}\left(0;\mu_{i},\theta \right), & \text{for}\;z_{i}=0\\ \left(1-\phi_{i}\right)f^{\text{NB}}\left(z_{i};\mu_{i},\theta \right), & \text{for}\;z_{i}>0 \end{array} \end{array}$$ (3)

where 0 < *ϕ*_*i*_ < 1 is the probability of an excess zero response. Here we do not consider a Poisson hurdle model or zero-inflated Poisson model. Those two models tend to have substantially inflated type I error [7]. This is due to the conditional variance being greater than the conditional mean, which violates the assumption of the Poisson model, especially for microbiome data.

We fit GLMM models with an R package called glmmTMB [22], which is available in CRAN.

#### Likelihood ratio test

In this paper, we applied the likelihood ratio test (LRT) to test whether the phenotype *Y* is associated with each OTU $Z_{i}^{\left(j\right)}$ for *j* = 1, …, *m* based on a GLMM model. In this section, we will briefly describe how to apply LRT to GLMM. A more detailed discussion of the LRT for testing fixed effects of GLMMs can be found in the work of Bolker et al. [23] and the references therein. We will omit OTU index *j* for simplicity. In LRT, we test the following two model assumptions:

$$\begin{array}{c} \begin{array}{cc} H_{0}:Z_{i} & ∼\;f(z_{i}|X_{i}^{\left(1\right)},\cdots ,X_{i}^{\left(s\right)},W_{i}^{\left(1\right)},\cdots ,W_{i}^{\left(t\right)}),\\ H_{1}:Z_{i} & ∼\;f(z_{i}|Y,X_{i}^{\left(1\right)},\cdots ,X_{i}^{\left(s\right)},W_{i}^{\left(1\right)},\cdots ,W_{i}^{\left(t\right)}) \end{array} \end{array}$$ (4)

Let *L*_0_ and *L*_1_ represent the maximized likelihoods under models *H*_0_ and *H*_1_ respectively. The log likelihood ratio statistic is defined as:

$$\begin{array}{c} T_{n}=2\left(\log L_{1}-\log L_{0}\right). \end{array}$$ (5)

By Wilk’s theorem [24], the sampling distribution of *T*_*n*_ under *H*_0_ is asymptotically a chi-square distribution with degrees of freedom *α*, which is equal to the difference of the numbers of parameters in *H*_0_ and *H*_1_. Suppose the number of levels of *Y* is *K*. Then the degrees of freedom *α* is equal to *K* − 1 in NB mixed model, and *α* is equal to 2(*K* − 1) in TPNB and ZINB if *Y* is used to model both *μ*_*i*_ and *ϕ*_*i*_.

#### False discovery rate and q-value

Due to the large number of OTUs, we need to convert p-values into FDR adjusted p-values, or q-values, to better understand the chance of false positives. The q-value of the *j*th test a p-value *p*^(*j*)^ is the FDR if we use *p*^(*j*)^ as the cutoff *t* in feature selection; i.e., features with p-values ≤*p*^(*j*)^ are selected. To ensure theoretical monotonicity, *q*(*p*^(*j*)^) is defined as the minimum of *FDR*(*t*) for *t* ≥ *p*^(*j*)^ [25]:

$$\begin{array}{c} q\left(p^{\left(j\right)}\right)=\min_{t\geq p^{\left(j\right)}}\hat{\text{FDR}}\left(t\right), \end{array}$$ (6)

where

$$\begin{array}{c} \hat{\text{FDR}}\left(t\right)=\frac{m\cdot {\hat{\pi }}_{0}\cdot t}{\#\{p^{j}\leq t\}}, \end{array}$$

and ${\hat{\pi }}_{0}$ is the estimated proportion of null hypothesis,

$$\begin{array}{c} \begin{array}{cc} & {\hat{\pi }}_{0}\left(\zeta \right)=\frac{\#\{p^{\left(j\right)}\geq \zeta ;j=1,...,m\}}{m(1-\zeta )},\\ & s.t.\;{\text{p-value}}_{p^{\left(j\right)}\geq \zeta }∼Unif\left(0,1\right). \end{array} \end{array}$$

If we order all the p-values, and denote the *j*th p-value by *p*^[*j*]^, an approximation for the q-value is $\hat{q}\left(p^{\left[j\right]}\right)=m\cdot {\hat{\pi }}_{0}\cdot p^{\left[j\right]}/j$, which may not be monotone with *p*^[*j*]^, but is easy to calculate and typically close to *q*(*p*^[*j*]^) when *m* is large; see a discussion of FDR by Yin et al. [26].

When the model for the response variable is correctly specified, the q-values are good estimates of the actual *FDR*s, as explained above. In such cases, the q-value is useful guidance for determining the cutoff *t* in feature selection. However, in practice, the correctness of a model often lacks serious verification. This problem is particularly crucial in microbiome data analysis because OTU counts are difficult to model due to clustering, over-dispersion, and zero-inflation.

### Predictive analysis methods

#### Transformation of OTU counts

Statistical learning is another important alternative approach to selecting OTUs. In microbiome data, it is often believed that the phenotype affects the composition of OTUs, rather than the raw counts of OTUs, which are also affected by the total reads *T*_*i*_. As such, a reasonable transformation for OTU counts is the variance-stability transformation of the proportion of the counts of the *j*th OTU among those of all OTUs:

$${\tilde{Z}}_{i}^{\left(j\right)}=\arcsin(\sqrt{Z_{i}^{\left(j\right)}/T_{i}}),\;\text{for}\;j=1,\ldots ,m$$

Other transformations can be investigated too. For example, if we believe that only the presence or absence of certain OTUs is related to the phenotype, we transform $Z_{i}^{\left(j\right)}$ by ${\tilde{Z}}_{i}^{\left(j\right)}=I\left(Z_{i}^{\left(j\right)}>0\right)$. This binary transformation is useful for eliminating the adverse effect of the over-dispersion in OTU counts.

To be consistent with conventional notations for statistical learning models, the predictor variables are collectively denoted by *x*_*i*_, which includes ${\tilde{Z}}_{i}^{\left(j\right)}$ and all other fixed and random factors (represented by binary indicator variables). The *x*_*i*_ will be a column vector in this section. The first value in *x*_*i*_ is “1” for including an intercept. After we fit a statistical learning model for *y*_*i*_ given *x*_*i*_, selection of OTUs can be achieved by looking at the coefficients associated with the transformed OTU variables ${\tilde{Z}}_{i}^{\left(j\right)}$.

#### LASSO multinomial logistic regression

LASSO adds the *L*_1_ of the regression coefficients as a penalty term to the log likelihood function to achieve shrinkage of regression coefficients for avoiding over-fitting and for achieving feature selection [16]. Suppose the response variable *y*_*i*_ has *K* levels, that is, *y*_*i*_ takes values in {1, 2, …, *K*}. The multinomial logistic regression links the probability of *y*_*i*_ = *k* to *x*_*i*_ using the soft-max function as follows:

$$\begin{array}{c} P\left(y_{i}=k|x_{i},\beta_{1},\ldots ,\beta_{K}\right)=\frac{\exp(\beta_{k}^{T}x_{i})}{\sum_{k=1}^{K}\exp(\beta_{k}^{T}x_{i})},\; \end{array}$$ (7)

for *k* = 1, …, *K*, where *β*_*k*_ is the collection of all regression coefficients related to *y*_*i*_ = *k*, which is a column vector of the same length of *x*_*i*_. We will denote all these regression coefficients collectively by *β*. Given observations {(*y*_*i*_, *x*_*i*_), *i* = 1, …, *n*} and a tuning parameter λ, the LASSO-penalized negative log likelihood function is

$$\begin{array}{c} l_{\text{LASSO}}\left(\beta \right)=-\sum_{i=1}^{n}\log\left(P\left(y_{i}|x_{i},\beta \right)\right)+\lambda \sum_{k=1}^{K}\left|\right|\beta_{k}{\left|\right|}_{1}, \end{array}$$ (8)

where ||*β*_*k*_||_1_ is the *L*_1_ penalty of *β*_*k*_, equal to the sum of absolute values in *β*_*k*_ (except the intercept). The LASSO estimate of *β* is the minimizer of the LASSO-penalized negative log likelihood function *l*_LASSO_(*β*).

*L*_1_ penalty can shrink the coefficients associated with less important predictor variables exactly into zeros. The OTU variables with non-zero coefficients will be selected. The degree of coefficient shrinkage is controlled by the tuning parameter λ. Larger λ enforces greater shrinkage to the coefficients *β*, resulting in selecting fewer predictors in *x*_*i*_. Therefore, the role of λ is similar to that of the cutoff *t* for thresholding p-values in statistical testing methods. In a predictive analysis, a straightforward method for choosing λ is to look at the predictive performance in test samples, which is measured by chosen metrics; for example, the error rate in predicting *y*_*i*_. Cross-validation (CV) is a procedure to split the dataset into artificial training and test sets to obtain out-of-sample predictive metrics. *K*-fold CV means that we randomly split the data into *k* folds of approximately equal size. The k-1 folds are used as training and the remaining 1 fold is used as validation. The optimal λ is chosen with smallest CV error rate.

#### LASSO-MLR with variable screening

Variable screening is a commonly used feature selection method for high dimensional data to reduce dimentionality. We conduct LRT for each feature on training data and convert the p-values calculated from LRT on training data to q-values. A subset of features *Z*^(1*)^, …, *Z*^(m*)^ is selected by thresholding q-values with a specific cutoff. LASSO is built based on *Z*^(1*)^, …, *Z*^(m*)^ and other covariates.

#### Predictive metrics

Let ${\hat{P}}_{i}\left(k|x_{i}\right)$ be a predictive probability of *y*_*i*_ = *k* for *k* = 1, …*K*. We can assess the goodness of $\hat{P}\left(y_{i}|x_{i}\right)$ with actually observed {*y*_*i*_;*i* = 1, …, *n*}. The first metric is error rate. We predict *y*_*i*_ by $\hat{y_{i}}={\mathrm{argmax}}_{k}\hat{P_{i}}\left(k|x_{i}\right).$ The error rate is defined as the proportion of wrongly predicted cases:

$$\begin{array}{c} ER=\frac{1}{n}\;\sum_{i=1}^{n}\;I\left({\hat{y}}_{i}\neq y_{i}\right). \end{array}$$

*ER* should be interpreted relatively, not absolutely. Let *f*_*k*_ be the observed frequency of *y*_*i*_ = *k* for *k* = 1, …, *K*. Without including any predictor in *x*_*i*_ (called the null model), the naive predictive probabilities are ${\hat{P}}_{i}^{\left(0\right)}\left(k\right)=f_{k}$ for *k* = 1, …, *K*. The actual CV predictive probabilities will estimate *f*_*k*_ with the *y*_*i*_ removed, but the frequency without considering *y*_*i*_ is very close to *f*_*k*_. Based on these naive predictive probabilities, the point prediction is that ${\hat{y_{i}}}^{\left(0\right)}={\mathrm{argmax}}_{k}f_{k}$ for *i* = 1, …, *n*. The *ER* with ${\hat{P}}_{i}^{\left(0\right)}\left(k\right)$ is *ER*^(0)^ = 1 − max{*f*_*k*_;*k* = 1, …, *K*}, which we will call the baseline error rate. Similar to *R*^2^ used in linear regression, a relative predictivity metric based on *ER* is defined as the percentage of the reduction of *ER* from *ER*^(0)^:

$$\begin{array}{c} R_{\text{ER}}^{2}=\frac{ER^{\left(0\right)}-ER}{ER^{\left(0\right)}}. \end{array}$$

$R_{\text{ER}}^{2}$ is a better metric to show the model performance when the dataset is highly unbalanced.

We use area under the (receiver operating characteristic) ROC curve (AUC) as the second metric. AUC represents a trade-off between sensitivity (true positive rate) and specificity (false positive rate). The latter are defined as:

$$\begin{array}{l} \text{sensitivity}=\frac{\#\text{of}\;\text{true}\;\text{positives}}{\#\text{of}\;\text{true}\;\text{positives}+\#\text{of}\;\text{false}\;\text{negatives}},\\ \text{specificity}=\frac{\#\text{of}\;\text{true}\;\text{negatives}}{\#\text{of}\;\text{true}\;\text{negatives}+\#\text{of}\;\text{false}\;\text{positives}}. \end{array}$$

Unlike error rate that requires a decision threshold (usually 0.5 is taken) to discriminate, AUC is independent of the decision threshold. It measures each possible performance as the decision threshold is varied. For every cutoff point *c* ∈ [0, 1], *y*_*i*_ is predicted by ${\hat{y}}_{i}=I\left({\hat{P}}_{i}\left(y_{i}|x_{i}\right)\geq c\right)$, where *I*(⋅) is the indicator function which is equal to 1 if the condition in the bracket is true, 0 otherwise. Sensitivity and specificity are determined by comparing ${\hat{y}}_{i}$ and the true label *y*_*i*_. The ROC curve is plotted based on the sensitivity and specificity of each cutoff point. AUC is the area under the ROC curve.

We also use area under the precision-recall (PR) curve (AUPRC) as the third predictive metric. Similar to ROC curve, PR curve is a curve of precision verses recall at varying cutoff points for predictive probabilities. Precision and recall are defined as:

$$\begin{array}{ll} \text{precision} & =\frac{\#\text{of}\;\text{true}\;\text{positives}}{\#\text{of}\;\text{true}\;\text{positives}+\#\text{of}\;\text{false}\;\text{positives}},\\ \text{recall} & =\frac{\#\text{of}\;\text{true}\;\text{positives}}{\#\text{of}\;\text{true}\;\text{positives}+\#\text{of}\;\text{false}\;\text{negatives}}. \end{array}$$

ROC curves and PR curves are widely used in unbalanced data. PR curves are also used for balanced dataset to highlight performance differences that are lost in ROC curves [27].

## Data

### Synthetic datasets

Synthetic datasets are generated to mimic the real microbiome data structure to assess the predictive performance of each method. Dirichlet-multinomial distribution has been shown as a good model to account for overdispersion of microbiome dataset [6]. We estimate the the parameters of the PD dataset that we use for real data analysis using Dirichlet-multinomial distribution. Then we generate the OTU counts based on Dirichlet-multinomial model given the estimated parameters. The total reads for each sample are sampled from *Unif*(10, 000, 30, 0000). The uniform distribution is a commonly used distribution to generate total reads [6]. The parameters of the uniform distribution is empirically estimated from the PD dataset. Besides OTUs, fixed factors *X*^(1)^ and *X*^(2)^, and random factor *W* are also generated. Phenotype *Y* is generated from logistic regression given OTUs, fixed and random factors. We will consider three different datasets with *n* samples and *m* OTUs, which are listed in Table 2. The number of OTUs *m* = 587 is the same for each dataset. The sample size *n* = 500 for dataset 1 and 2, *n* = 262 for dataset 3 to mimic the sample size of real data. Dataset 1 and 3 include random effect while dataset 2 does not include random effect when generating phenotype *Y*. We generate 100 datasets using each of the three schemes. The details of data generation are described as follows:

- Randomly select 20 OTUs out of 587 OTUs as true signals. The parameters $\beta_{Z}^{i^{*}}$ are sampled from *Unif*(1.5, 2) for i* = 1, …, 10 and *Unif*(−2, −1.5) for i* = 11, …, 20 to ensure the balance of phenotype. The parameters for all the other OTUs are set to be 0.
- Generate two fixed factors *X*^(1)^ and *X*^(2)^. *X*^(1)^ is a continuous variable (such as age), where *X*^(1)^ ∼ *Unif*(20, 50), *β*_*X*^(1)^_ = −1. *X*^(2)^ is a three level categorical variable, and *β*_*X*^(2)^_ = (10, 15) to balance the negative effect of *X*^(1)^ to phenotype.
- In dataset 1 and 3, a random factor *W* is generated. We divide the *n* samples into 5 groups (e.g., country), denoted as *W*. *β*_*W*_ = (−100, −50, 0, 50, 100) represents effect of each group. The gap of each group is set to be 50 to make sure the scale is similar to simulated OTU.
- *Y* is generated by *Y* ∼ Bernoulli(*p*), where,
  $$\begin{array}{c} p=1/(1+\exp\left(\beta_{0}+Z\beta_{Z}+X\beta_{X}+W\beta_{W}+\epsilon \right)), \end{array}$$ (9)
  for dataset 1 and 3, and
  $$\begin{array}{c} p=1/(1+\exp\left(\beta_{0}+Z\beta_{Z}+X\beta_{X}+\epsilon \right), \end{array}$$ (10)
  for dataset 2, where *β*_0_ = 1 and *ϵ* ∼ *N*(0, 10).

**Table 2:** The setting of each synthetic dataset.

Dataset	sample size n	number of OTUs m	random effect *W*
Dataset 1	500	587	✓
Dataset 2	500	587	
Dataset 3	262	587	✓

### A human gut microbiome dataset related to PD

Parkinson’s disease (PD) is a progressive nervous system disorder that affects movement. The etiology of PD is still unknown, although researches have identified genetic loci, genes, and other environmental factors that are related to PD [28]. Evidence linking PD to the gut precedes the recent appreciation of the microbiome. Gastrointestinal symptoms often precede the motor signs of PD. Recently, there are a number of studies conducted to study the connection between the gut microbiome and PD [3, 29–33]. We use a dataset released by Hill-Burns et al. [30]. A total of 327 subjects, comprising 197 cases (60%) of PD and 130 controls (40%), were enrolled in the study cohort to investigate the association of the dysbiosis of the gut microbiome and potential confounders of PD. Stool samples were collected for DNA extraction and 16S rRNA amplicon sequencing. Sequencing was performed using an Illumina MiSeq. OTUs were picked in QIIME using the August 2013 release of the Greengenes 16S rRNA gene sequence database as reference at 97% similarity.

Hill-burns et al. [30] applied conventional statistical testing methods based on GLMMs to the dataset and showed that the associations between PD and some gut microbial taxa are statistically significant. Statistical significance does not necessarily imply practical significance. We apply the three strategies of predictive analysis methods described above to measure the strength of the association by examining the predictability of PD given the OTUs and some covariates. In our analysis, we apply more stringent quality control in the microbiome data. Subjects with fewer than 10,000 reads and OTUs with a prevalence less than 20% are removed for controlling data quality. The final dataset consists of *n* = 262 subjects, in which there are 160 cases (61%) of PD and 102 controls (39%). The proportion of PD in this subset is nearly the same as in the 327 subjects. 587 non-redundant bacterial taxa are retained after quality filtering. These taxa are comprised of 42 orders, 227 families, 245 genera, and 73 species. The mean age of the cohort is 69.13±9.04(*SD*) years (range of 44-94 years), and 45% of the cohort is female.

## Results and discussions

### Results of analyzing synthetic datasets

In this section, we investigate LASSO, screening+GLM, and screening+LASSO on the synthetic datasets. We split each dataset into a training set with 80% subjects and a test dataset with the remaining 20% subjects. Only the training set is used to select features (OTUs) and to train the GLM or LASSO-MLR models. The test set is used to check the predictive performance of selected features through GLM or LASSO-MLR. We also investigate whether random effect will help to improve the predictive performance. Therefore, the analysis of dataset 1 includes two parts: part 1 is to include random effect *W* in screening and prediction; part 2 is to exclude random effect *W* in screening and prediction. The analysis of dataset 2 is similar to part 2 of dataset 1 and the analysis of dataset 3 is the same as part 1 of dataset 1.

In statistical testing, we are interested in whether the presence or absence of phenotype will affect the reads of OTU counts. Hence, we treat phenotype *Y* as fixed effect in fitting the GLMM model. We conduct the screening by using LRT to test whether phenotype *Y* has an effect on each OTU. We drop the OTUs which have zero proportion (ZP) greater than 90% by setting the p-values of those OTUs to be 1. A vector of *m* = 587 p-values is calculated for each model and each dataset. Table 3 shows the average number of true signals selected by each model and the power under a cut of q-value of 0.05 over 100 iterations. We can see that TPNB model can select the most of the true signals, it also has higher power than ZINB and NB model. The performance of ZINB and NB model is similar. It is of interest to notice that when we exclude random effect in part 2 of dataset 1, the power of all the three methods increases significantly. When the sample size decrease in dataset 3, the power of each method also drops. This shows that the screening method is drastically affected by the sample size and whether random effects are considered.

**Table 3:** The average number of included true signals and power for each method under a cut of q-value of 0.05.

Model	Average number of included true signals				Power			
Dataset	1 (part1)	1 (part2)	2	3	1 (part1)	1 (part2)	2	3
ZINB	7.090	7.430	7.920	2.400	0.388	0.418	0.444	0.156
TPNB	11.200	11.380	11.550	4.910	0.644	0.657	0.663	0.336
NB	7.790	7.930	8.110	2.480	0.405	0.414	0.423	0.134

We implement three strategies of predictive analysis on our simulated datasets: (1) **LASSO**: fitting LASSO-MLR using the factors *X*^(1)^, *X*^(2)^, and *W* if applicable, and all 587 transformed OTU variables as predictors; (2) **screening+GLM**: conducting variable screening on all the 587 OTUs using LRT methods applied to GLMMs, then fitting a GLM using fixed and random effects and a selected subset of OTUs as predictors; (3) **screening+LASSO**: conducting variable screening and then fitting LASSO to fixed and random effects and a selected subset of OTUs as predictors. 200 values of λ are chosen in a reasonable range for shrinking coefficients towards zeros and thus to select OTUs. The choice of λ is guided by predictive metrics estimated from the 10-fold CV in the training dataset. We transform OTU counts through the variance-stability transformation and binary transformation as described above. For variable screening, we use the R function p.adjust from the package stats to convert p-values into q-values. Calculating q-value is a method for estimating actual FDRs given only p-values. We choose the conservative method called “BH” [34].

Table 4 shows the predictive performance of each approach based on variance-stability transformed OTU counts for dataset 1. We calculate the mean and standard deviation (SD) of predictive metrics over 100 iterations. For comparison, it also shows the predictive performance of the oracle method, which fits a GLMM given the 20 truly related OTUs, 2 fixed factors *X*^(1)^, *X*^(2)^, and random effect *W* as predictors. The ER of oracle case is 0.04. To better understand the predictive metrics, we mention that the naive prediction without considering any predictor will give an error rate of 0.4 for dataset 1. We can see that LASSO without variable screening performs remarkably well on dataset 1 based on variance-stability transformation. The ER of LASSO is 0.21 ($R_{ER}^{2}=0.475$) and both the AUC and AUPRC are 0.87. The features selected by LASSO include 20 true signals, which shows LASSO is able to identify the truly related features when there are signal predictors for the response. The screening+GLM and screening+LASSO methods also work well for these datasets when the screening is based on TPNB models, with predictive metrics close to those of LASSO. This is due to the high power of TPNB. The SDs of AUC and AUPRC of LASSO are smaller than that of TPNB+GLM and TPNB+LASSO, which shows LASSO is more stable over 100 iterations. It is interesting to notice that when we exclude the random effect from each model, the ER of all methods increase by 0.01, and the AUC and AUPRC decrease by 0.01. However, Table 3 shows that the power increases without considering random effect. This result indicates that random effect can help to increase the predictive performance when random effect really exists. Table 5 shows the performance of each approach based on binary transformed OTUs for dataset 1. In this case, LASSO is not able to improve the predictive performance from baseline. ERs of Screening+GLM or Screening+LASSO increase by 0.02 or 0.03 from baseline ER of 0.4. This shows that binary transformation is not an appropriate transformation for this dataset.

**Table 4:** Predictive performance of different approaches based on the variance-stability transformation for dataset 1.

	Dataset 1, including random effect *W*			Dataset 1, excluding random effect *W*		
Method	ER (SD)	AUC (SD)	AUPRC (SD)	ER (SD)	AUC (SD)	AUPRC (SD)
Oracle	0.04 (0.02)	0.98 (0.02)	0.97 (0.03)	0.04 (0.02)	0.98 (0.02)	0.32 (0.1)
LASSO	**0.21 (0.05)**	**0.87 (0.04)**	**0.87 (0.07)**	0.22 (0.05)	0.86 (0.05)	0.86 (0.07)
ZINB+GLM	0.24 (0.06)	0.82 (0.06)	0.82 (0.09)	0.25 (0.06)	0.81 (0.07)	0.81 (0.09)
TPNB+GLM	**0.2 (0.05)**	**0.87 (0.05)**	**0.86 (0.08)**	0.21 (0.05)	0.86 (0.05)	0.85 (0.09)
NB+GLM	0.23 (0.05)	0.83 (0.05)	0.83 (0.08)	0.24 (0.06)	0.82 (0.06)	0.82 (0.09)
ZINB+LASSO	0.24 (0.06)	0.82 (0.06)	0.82 (0.09)	0.25 (0.06)	0.81 (0.07)	0.81 (0.09)
TPNB+LASSO	**0.2 (0.05)**	**0.87 (0.05)**	**0.86 (0.08)**	0.21 (0.05)	0.86 (0.05)	0.85 (0.09)
NB+LASSO	0.23 (0.05)	0.83 (0.05)	0.83 (0.08)	0.24 (0.06)	0.82 (0.06)	0.82 (0.09)

**Table 5:** Predictive performance of different approaches based on the binary transformation for dataset 1.

	Dataset 1, including random effect *W*			Dataset 1, excluding random effect *W*		
Method	ER (SD)	AUC (SD)	AUPRC (SD)	ER (SD)	AUC (SD)	AUPRC (SD)
LASSO	0.43 (0.06)	0.56 (0.06)	0.56 (0.13)	0.44 (0.06)	0.56 (0.05)	0.55 (0.13)
ZINB+GLM	0.38 (0.06)	0.62 (0.06)	0.62 (0.12)	0.39 (0.06)	0.6 (0.06)	0.61 (0.12)
TPNB+GLM	0.38 (0.06)	0.63 (0.07)	0.63 (0.13)	0.38 (0.06)	0.62 (0.06)	0.62 (0.13)
NB+GLM	0.37 (0.06)	0.63 (0.07)	0.64 (0.12)	0.38 (0.06)	0.62 (0.07)	0.62 (0.13)
ZINB+LASSO	0.37 (0.06)	0.63 (0.06)	0.63 (0.12)	0.39 (0.06)	0.6 (0.06)	0.61 (0.12)
TPNB+LASSO	0.38 (0.06)	0.63 (0.07)	0.63 (0.13)	0.38 (0.06)	0.62 (0.06)	0.62 (0.13)
NB+LASSO	0.37 (0.06)	0.64 (0.06)	0.64 (0.13)	0.38 (0.06)	0.62 (0.07)	0.62 (0.13)

Table 6 shows the predictive performance of each method based on variance-stability transformation for dataset 2 and 3. Dataset 2 is generated without random effect. LASSO, TPNB+GLM, TPNB+LASSO still outperform other methods. In dataset 3 with only around half of the sample size, LASSO performs better over other methods. The average number of true signals selected by LASSO over 100 iterations is 16. This shows that when the sample size decreases, LASSO is still able to detect 80% of true signals. However, the average number of true signals selected by screening are less than 5 as shown in Table 3. The predictive performance of screening+GLM and screening+LASSO will be highly affected by the performance of screening since the power of test will be affected by sample size and the model we use. Table 7 shows that the the binary transformation still does not work well for this dataset. These results show that LASSO has very stable and excellent performance on each dataset under the variance-stability transformation. The random effect can help to improve the predictive performance and an appropriate transformation is crucial for achieving good predictions with such zero-inflated and over-dispersed count data.

**Table 6:** Predictive performance of different approaches based on the variance-stability transformation for dataset 2 and 3.

	Dataset 2			Dataset 3		
Method	ER (SD)	AUC (SD)	AUPRC (SD)	ER (SD)	AUC (SD)	AUPRC (SD)
Oracle	0.04 (0.02)	0.97 (0.02)	0.97 (0.03)	0.08 (0.04)	0.96 (0.04)	0.94 (0.07)
LASSO	**0.2 (0.04)**	**0.87 (0.04)**	**0.87 (0.06)**	**0.28 (0.07)**	**0.79 (0.07)**	**0.79 (0.1)**
ZINB+GLM	0.23 (0.05)	0.84 (0.06)	0.84 (0.08)	0.36 (0.08)	0.68 (0.11)	0.68 (0.15)
TPNB+GLM	**0.19 (0.05)**	**0.88 (0.04)**	**0.88 (0.06)**	**0.3 (0.08)**	**0.75 (0.09)**	**0.74 (0.13)**
NB+GLM	0.23 (0.05)	0.84 (0.06)	0.84 (0.08)	0.36 (0.08)	0.68 (0.11)	0.68 (0.15)
ZINB+LASSO	0.23 (0.05)	0.84 (0.06)	0.84 (0.08)	0.34 (0.08)	0.69 (0.1)	0.69 (0.15)
TPNB+LASSO	**0.19 (0.05)**	**0.88 (0.04)**	**0.88 (0.06)**	**0.3 (0.07)**	**0.75 (0.09)**	**0.75 (0.12)**
NB+LASSO	0.23 (0.05)	0.84 (0.05)	0.84 (0.08)	0.34 (0.07)	0.7 (0.09)	0.7 (0.14)

**Table 7:** Predictive performance of different approaches based on the binary transformation for dataset 2 and 3.

	Dataset 2			Dataset 3		
Method	ER (SD)	AUC (SD)	AUPRC (SD)	ER (SD)	AUC (SD)	AUPRC (SD)
LASSO	0.44 (0.05)	0.56 (0.05)	0.56 (0.12)	0.45 (0.07)	0.56 (0.06)	0.55 (0.13)
ZINB+GLM	0.39 (0.05)	0.61 (0.06)	0.62 (0.11)	0.42 (0.08)	0.58 (0.07)	0.59 (0.13)
TPNB+GLM	0.39 (0.06)	0.62 (0.07)	0.63 (0.12)	0.41 (0.08)	0.59 (0.07)	0.6 (0.13)
NB+GLM	0.38 (0.05)	0.62 (0.06)	0.62 (0.12)	0.41 (0.07)	0.59 (0.07)	0.6 (0.13)
ZINB+LASSO	0.39 (0.05)	0.61 (0.06)	0.62 (0.11)	0.41 (0.07)	0.59 (0.07)	0.6 (0.13)
TPNB+LASSO	0.39 (0.06)	0.62 (0.07)	0.63 (0.12)	0.4 (0.07)	0.59 (0.08)	0.61 (0.13)
NB+LASSO	0.38 (0.05)	0.62 (0.06)	0.62 (0.12)	0.41 (0.07)	0.59 (0.08)	0.61 (0.13)

### Results of analyzing the gut microbiome data

In this section, we apply the three approaches from simulation to the human gut microbiome dataset released by Hill-Burns et al. [30]. The sample size *n* is 262. The total number of OTUs is 587. This is a binary classification problem with PD as the response.

**Fig 1:**
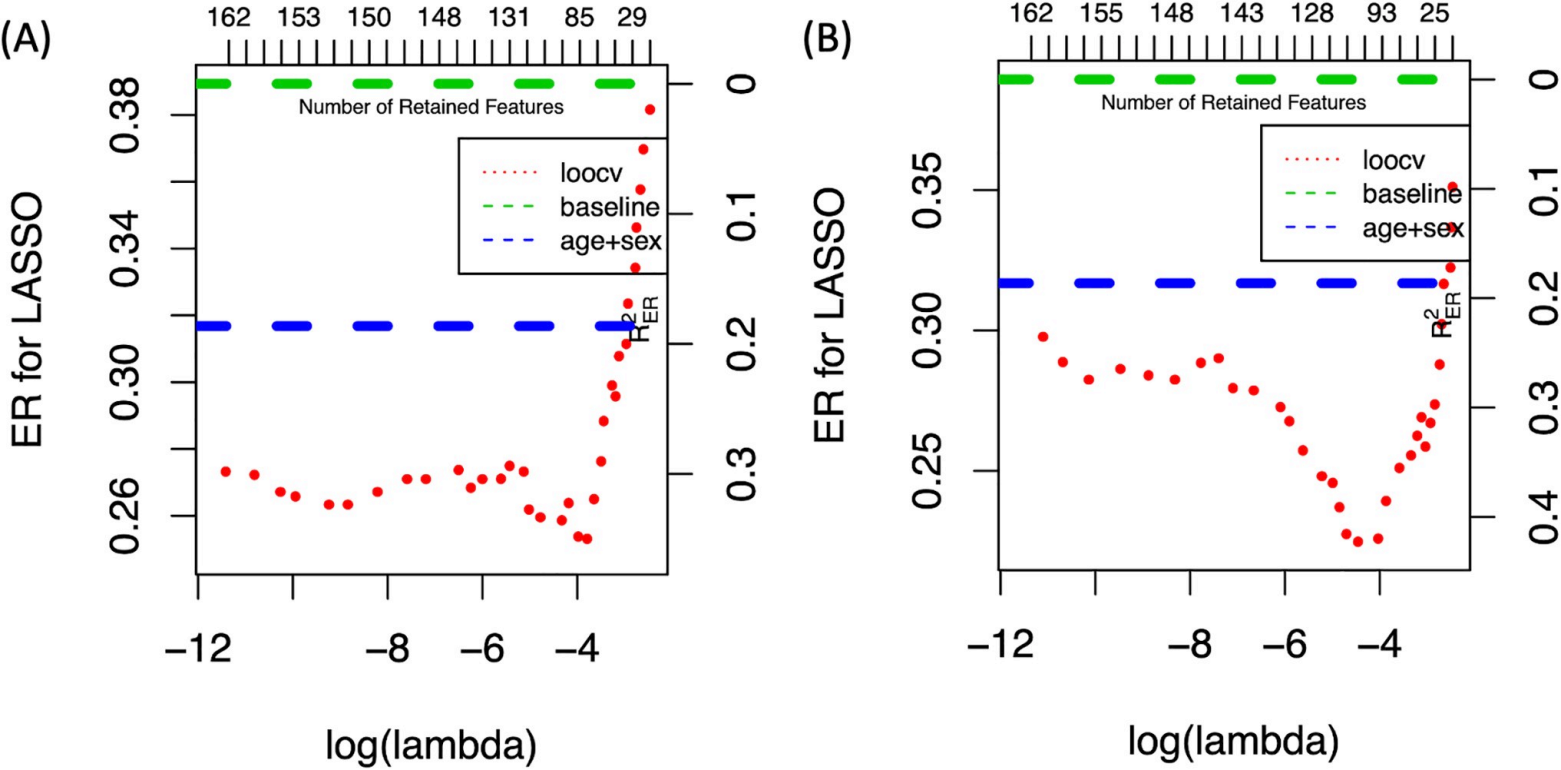
Predictive performance of LASSO under different choices of λ A: Error rate of LASSO based on variance-stability transformation with different choices of λ. B: Error rate of LASSO based on binary transformation with different choices of λ.

We first fit LASSO using Leave-one-out-cross-validation (LOOCV). We also implement LOOCV within the training samples in each fold to choose the optimal λ from 200 values of λ, which is the same set of λ used in analyzing the simulated datasets. The variance-stability transformation and binary transformation are exploited to transform OTUs. We consider models with different covariates, null (which means we only use transformed OTUs as predictors) and age. Table 8 shows that by treating age and sex as covariates, the model performances are improved significantly. Fig 1 shows how the error rate of predicting PD based on variance-stability and binary transformation changes with different values of λ. The green dashed line is the baseline and the blue dashed line shows the error rate of fitting the GLM model with only age and sex as predictors. For variance-stability transformation, the optimal error rate of 0.248 is achieved by including 83 OTUs, and age and sex as covariates. The optimal error rate of 0.221 by binary transformation is achieved by including 97 OTUs as predictors, and age and sex as covariates. This error rate reduces from the baseline error rate (0.39, the proportion of control patients in samples) by 43% ($R_{ER}^{2}=0.43$), and reduces by 36% from the error rate of GLM model with only age and sex as predictors.

**Table 8:** LASSO predictive performance based on the optimal choice of λ.

covariate (OTU transformation)	ER	AUC	AUPRC
null (variance-stability)	0.309	0.736	0.804
null (binary)	0.275	0.750	0.818
age (variance-stability)	0.260	0.785	0.844
age (binary)	0.256	0.794	0.855
age and sex (variance-stability)	0.248	0.811	0.872
age and sex (binary)	0.221	0.812	0.856

Then we implement the screening+GLM and screening+LASSO methods on this dataset using an external LOOCV method [35], in which the variable screening is re-done in each fold with only the training samples. For variable screening we fit three GLMM models (ZINB, TPNB, and NB) on each OTU by treating “PD”, “age”, and “sex” as fixed effects. We conduct the LRT on each OTU to see whether it is affected by PD. Then we convert the p-values into the q-values to look at the chance of each OTU being a false positive. The average numbers of OTUs selected from ZINB, TPNB, and NB models across all the folds are 100, 119, and 63, respectively by thresholding q-values with 0.05. Table 9 shows the predictive performance of the three approaches. We can see that binary transformation works better for this dataset. The optimal accuracy is achieved by ZINB+LASSO with ER = 0.199 based on binary transformation, followed by TPNB+LASSO with ER = 0.218. The AUCs for LASSO, ZINB+LASSO, TPNB+LASSO are above 0.8 and the AUPRCs are above 0.85 based on binary transformation. These numbers provide a strong evidence to support the claim that there is a connection between PD and the gut microbiome. The poor performance of screening+GLM shows that there are a number of false positives. LASSO can further shrink the coefficients of false positives to 0.

**Table 9:** Predictive performance of different approaches based on the variance-stability transformation and the binary transformation for the gut microbiome data related to PD.

Dataset	Variance-stability transformation			Binary transformation		
Method	ER	AUC	AUPRC	ER	AUC	AUPRC
LASSO	**0.248**	**0.811**	**0.872**	**0.221**	**0.812**	**0.856**
ZINB+GLM	0.291	0.720	0.771	0.318	0.706	0.758
TPNB+GLM	0.352	0.663	0.725	0.318	0.703	0.754
NB+GLM	0.268	0.743	0.765	0.264	0.758	0.768
ZINB+LASSO	0.249	0.801	0.861	**0.199**	**0.872**	**0.912**
TPNB+LASSO	**0.256**	**0.810**	**0.873**	**0.218**	**0.853**	**0.890**
NB+LASSO	0.222	0.810	0.871	0.249	0.787	0.818

## Discussion and conclusions

In this paper, we conduct empirical studies using synthetic datasets and a real dataset to investigate the performance of three strategies of predictive analysis for zero-inflated microbiome data. Our studies with the three synthetic datasets show that the predictive performance of LASSO is remarkably excellent for zero-inflated data. The screening+GLM and screening+LASSO methods also work reasonably well if the screening is based on an appropriate model for the OTU counts, especially when the power of test is high. However, screening is highly related to the sample size and the model we choose. TPNB model has higher power than ZINB and NB model. Thus, TPNB+GLM and TPNB+LASSO have lower ER and higher AUC and AUPRC compared to ZINB and NB model. The predictive performance of LASSO is much better than screening+GLM and screening+LASSO when the sample size is small. Moreover, ignoring random effect will also affect the predictive performance if random effect exists.

Compared to some statistical learning methods that have been developed for microbiome data recently, LASSO with an appropriate transformation is a simple, robust, and effective method. For example, Li et al. [36] proposed a linear log-contrast model with a penalty term for microbiome data to predict continuous outcome (e.g. BMI). Other methods, such as using phylogenetic-tree guided penalty term [37, 38] or using an inverse regression model [39] to predict outcomes with count microbiome data. Those methods make use of the cluster structure among OTUs to improve the prediction. However, implementing their methods requires tuning a large number of parameters.

The applications of the three strategies of predictive analysis methods in the PD microbiome dataset show that LASSO can predict PD with microbial composition and two covariates (age and sex) accurately with error rates near 0.2 and AUCs higher than 0.8. The best predictive accuracy for PD with this dataset is obtained with a ZINB+LASSO method, which gives predictive metrics as follows: ER = 0.199, AUC = 0.872, AUPRC = 0.912. Our predictive analysis results provide strong evidences of the connection between PD and the gut microbiome. Despite this strong connection, we clarify that the good predictive accuracies for PD with microbial composition do not necessarily imply that PD is caused by the differentiation of the gut microbial composition between PD and control patients. We notice that it is also possible that the differentiation of the gut microbial composition is caused by the change of the life style (such as dietary choice) of PD patients. In the future, we will continue to explore the etiology of PD.

Although variance-stability transformation works consistently better than binary transformation in the simulation studies. In the real data analysis, binary transformation has better predictive performance than variance-stability transformation. This may be related to the zero proportion of related OTUs or the effect of count data to the phenotype. We will investigate which transformation works better in different conditions in the future.

In a nutshell, we present that LASSO is a simple and robust method for zero-inflated microbiome data. Appropriate transformation is crucial for LASSO. Screening can be used to reduce the number of OTUs. However, screening+GLM or screening+LASSO only works well when the power of test of screening method is high. In the meantime, random effects can improve the predictive performance. They should be included if they are suspected to be relevant to the phenotype.
